# Comparative genome analysis provides deep insights into *Aeromonas hydrophila* taxonomy and virulence-related factors

**DOI:** 10.1186/s12864-018-5100-4

**Published:** 2018-09-26

**Authors:** Furqan Awan, Yuhao Dong, Jin Liu, Nannan Wang, Muhammad Hassan Mushtaq, Chengping Lu, Yongjie Liu

**Affiliations:** 1 Joint International Research Laboratory of Animal Health and Food Safety, College of Veterinary Medicine, Nanjing Agricultural University, Nanjing, China; 2 Department of Epidemiology and Public Health, University of Veterinary and Animal Sciences, Lahore, Pakistan

**Keywords:** *Aeromonas*, Pan-genome, Mislabelled strains, Prophages, Antibiotic resistance patterns, Virulence factors

## Abstract

**Background:**

*Aeromonas hydrophila* is a potential zoonotic pathogen and primary fish pathogen. With overlapping characteristics, multiple isolates are often mislabelled and misclassified. Moreover, the potential pathogenic factors among the publicly available genomes in *A. hydrophila* strains of different origins have not yet been investigated.

**Results:**

To identify the valid strains of *A. hydrophila* and their pathogenic factors, we performed a pan-genomic study. It revealed that there were 13 mislabelled strains and 49 valid strains that were further verified by Average nucleotide identity (ANI), digital DNA-DNA hybridization (dDDH) and in silico multiple locus strain typing (MLST). Multiple numbers of phages were detected among the strains and among them Aeromonas phi 018 was frequently present. The diversity in type III secretion system (T3SS) and conservation of type II and type VI secretion systems (T2SS and T6SS, respectively) among all the strains are important to study for designing future strategies. The most prevalent antibiotic resistances were found to be beta-lactamase, polymyxin and colistin resistances. The comparative analyses of sequence type (ST) 251 and other ST groups revealed that there were higher numbers of virulence factors in ST-251 than in other STs group.

**Conclusion:**

Publicly available genomes have 13 mislabelled organisms, and there are only 49 valid *A. hydrophila* strains. This valid pan-genome identifies multiple prophages that can be further utilized. Different *A. hydrophila* strains harbour multiple virulence factors and antibiotic resistance genes. Identification of such factors is important for designing future treatment regimes.

**Electronic supplementary material:**

The online version of this article (10.1186/s12864-018-5100-4) contains supplementary material, which is available to authorized users.

## Background

*Aeromonas hydrophila* is an important emerging zoonotic pathogen of aquatic origin [1]. In humans, *A. hydrophila* can cause gastroenteritis, necrotizing fasciitis, septicaemia and meningitis [2]. It has multifactorial virulence factors, such as type III, type IV and type VI secretion systems (T3SS, T4SS and T6SS, respectively), exotoxins and endotoxins [3]. Moreover, its ability to produce biofilms also threatens the food industry [4]. Although once considered to be susceptible to quinolones, *A. hydrophila* is developing resistance against these antibiotics according to recent surveys [5]. Because of its ubiquitous nature, the strain-based pathogenicity and virulence of *A. hydrophila* are highly variable [6, 7]. Additionally, characteristics of various strains isolated from humans have not been extensively explored and categorized.

*Aeromonas* species have dynamic characteristics and are hard to classify into defined taxonomic groups [1]. This overlapping classification has caused a great deal of ambiguity among *A. hydrophila* strains [3]. For example, the strains SSU, Ah-3 and BWH65 were first characterized as *A. hydrophila*, but later bioinformatics analyses differentiated them and they were assigned to different taxa [2, 8, 9]. Traditional classification of *A. hydrophila* is based on multiple hybridization groups (HGs), 16S rRNA sequencing and multiple locus sequence type (MLST) [1]. Further, classification of *A. hydrophila* has also been proposed on the basis of the presence of specific virulence factors, but this character is highly strain-specific and may not be reliable [3]. Whole genome sequencing (WGS) is now being employed for routine surveillance and for detection of possible outbreaks due to its low cost, less cumbersome protocols and reduced time investment [10]. Based on WGS, core genome MLST (cgMLST), genome-to-genome distance calculations based on digital DNA-DNA hybridization (dDDH) and average nucleotide identity (ANI) have been introduced to classify and identify the isolates [11, 12]. Recently, the pan-genome and core genome based on WGS have been utilized to understand the typing of isolates [13]. This technique is also being used to identify mutations and microevolution among the constantly evolving genes [14]. Identification and distribution of virulence factors, bacteriophages, and antibiotic resistance genes among geographically isolated strains are important features of WGS [15]. Application of WGS to epidemiology has created an opportunity to correctly diagnose disease caused by *A. hydrophila* and has been helpful in outbreak investigation [10].

The aim of this study was to identify the core genome and to estimate the variation within the pan-genome of publicly available *A. hydrophila* genomes from NCBI. Further, these results could be helpful in identifying mislabelled or wrongly classified strains. Additionally, patterns of virulence factors, antibiotic resistance genes and bacteriophages were identified. The results form the basis for classifying *A. hydrophila* along with determining the epidemiological significance of virulence factors.

## Results

### Core and pangenome analysis of 62 strains

**Fig. 1:**
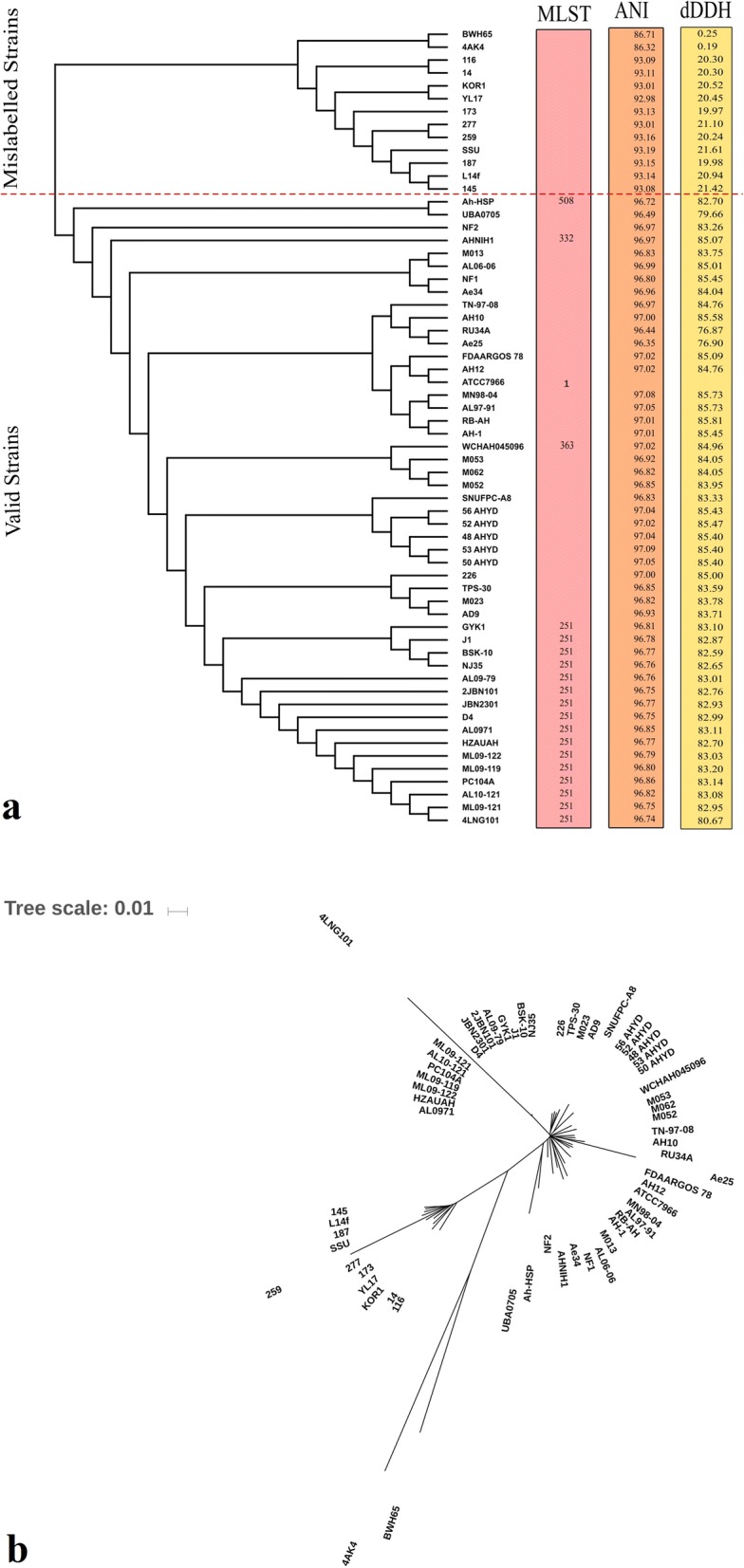
Core genome-based phylogeny: **a** The core genome-based phylogeny was verified by in silico MLST, ANI and dDDH. The red dotted line represents the cut-off line that separates the 13 mislabelled strains based on ANI and dDDH results. The remaining 49 strains are considered to be valid strains. **b** Core genome-based un-rooted phylogenetic tree. All the mislabelled strains are shown to be distinct from other valid strains. The remaining 49 strains are closely related to each other

It was revealed that there were 13 mislabelled strains included in the pan-genome of *A. hydrophila* (Fig. 1a). The remaining 49 strains were considered as valid members of the *A. hydrophila* pan-genome. A core gene tree was constructed based on the core genome (Fig. 1a and b). Among all the strains, several recently reported distinct strains, such as 4AK4, SSU and BWH65, were also included to observe their behaviours. Two of these strains, SSU and BWH65, were previously included in *A. hydrophila* but were later classified into *Aeromonas dhakensis* and *Aeromonas caviae*, respectively. These two strains were found to be distinct compared to the other strains, hence forming a distinct phylogenetic group as seen in Fig. 1a and b. To identify and verify this core genome-based tree, in silico MLST against the *Aeromonas* pubMLST database was performed by uploading the genomes to the CGE webserver. However, in silico MLST could identify some ST groups, while most of the genomes remained as unknown or nontypeable. For further identification, the ANI percentage and digital DNA-DNA hybridization (dDDH) were determined in comparison to the *A. hydrophila* ATCC7966 reference genome. Strains with greater than 95% ANI were considered to be the same species, while strains with dDDH values of more than 70% were considered to be same species. Figure 1a shows that ANI and dDDH results were consistent with each other and identified the strains closely related to SSU and BWH65 as distinct strains. Hence, the core genome phylogeny verified the presence of other strains in this pan-genome.

**Fig. 2:**
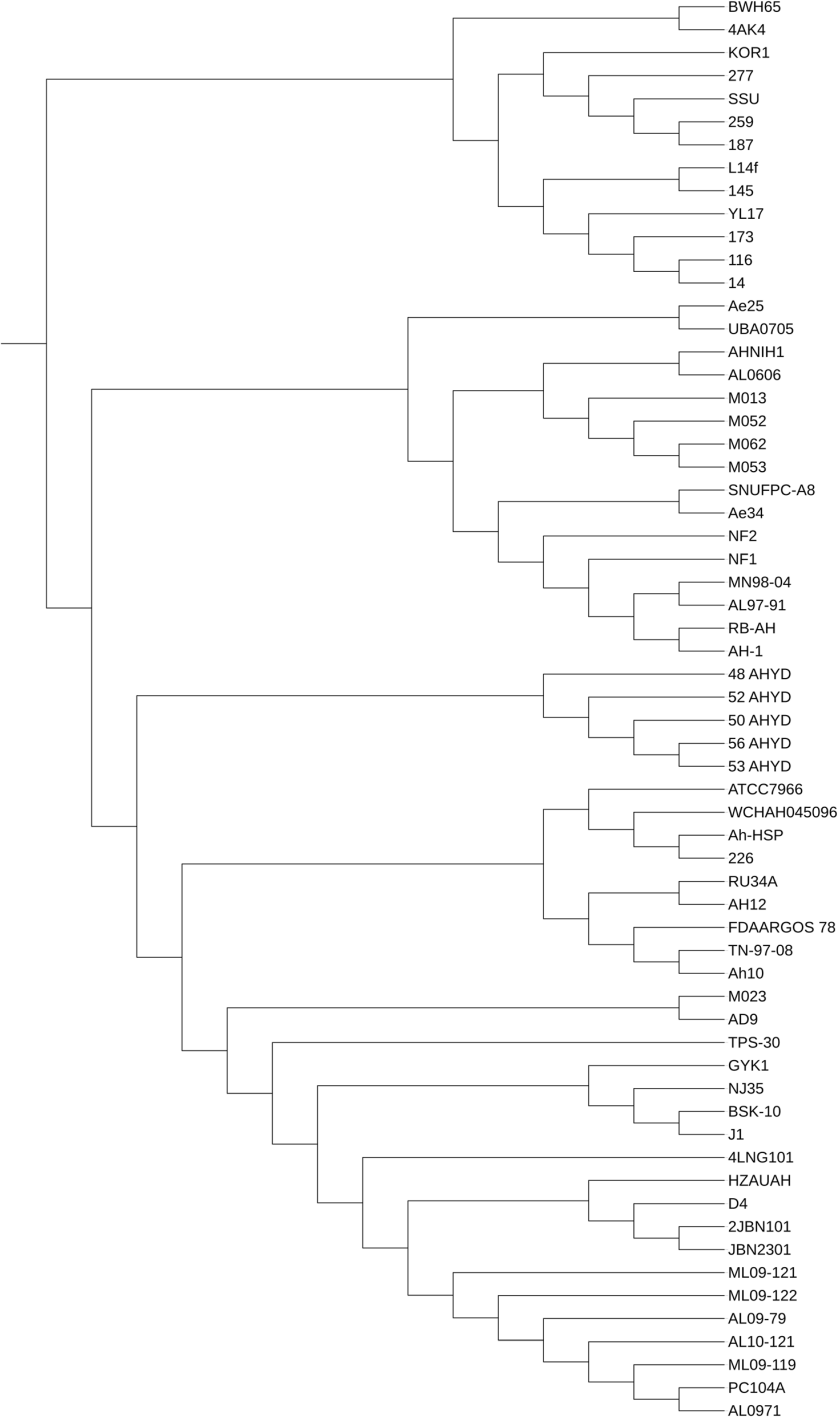
Pan-genome tree of 62 strains based on the presence or absence of genes with the mislabelled 13 strains represented as a separate group

A pan-genome phylogenetic tree based on the presence or absence of genes was also constructed (Fig. 2). All the strains that were closely related to SSU and BWH65 showed the same phylogeny, as expected. There was not much difference when the pan-genome tree was compared with the core genome tree. All the distinct strains were closely related to SSU and BWH65, as they were grouped in the core genome phylogeny tree.

### Characteristics of the valid 49 *A. hydrophila* genomes

**Fig. 3:**
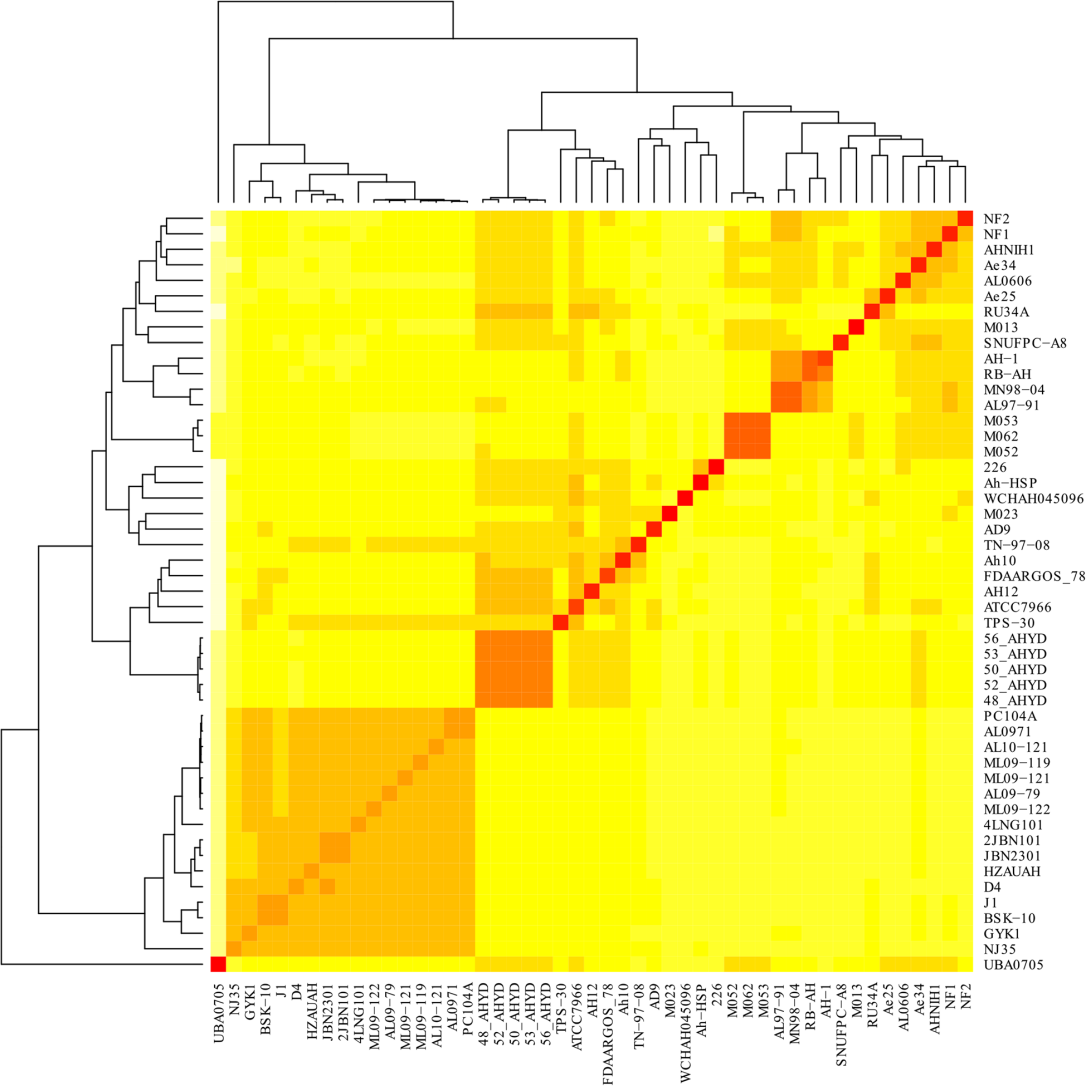
Heatmap chart generated from distances calculated based on the presence or absence of genes in all valid 49 strains. All ST-251 strains are grouped into one clade, while the UBA0705 strain from Australia showed little relation to other strains

**Fig. 4:**
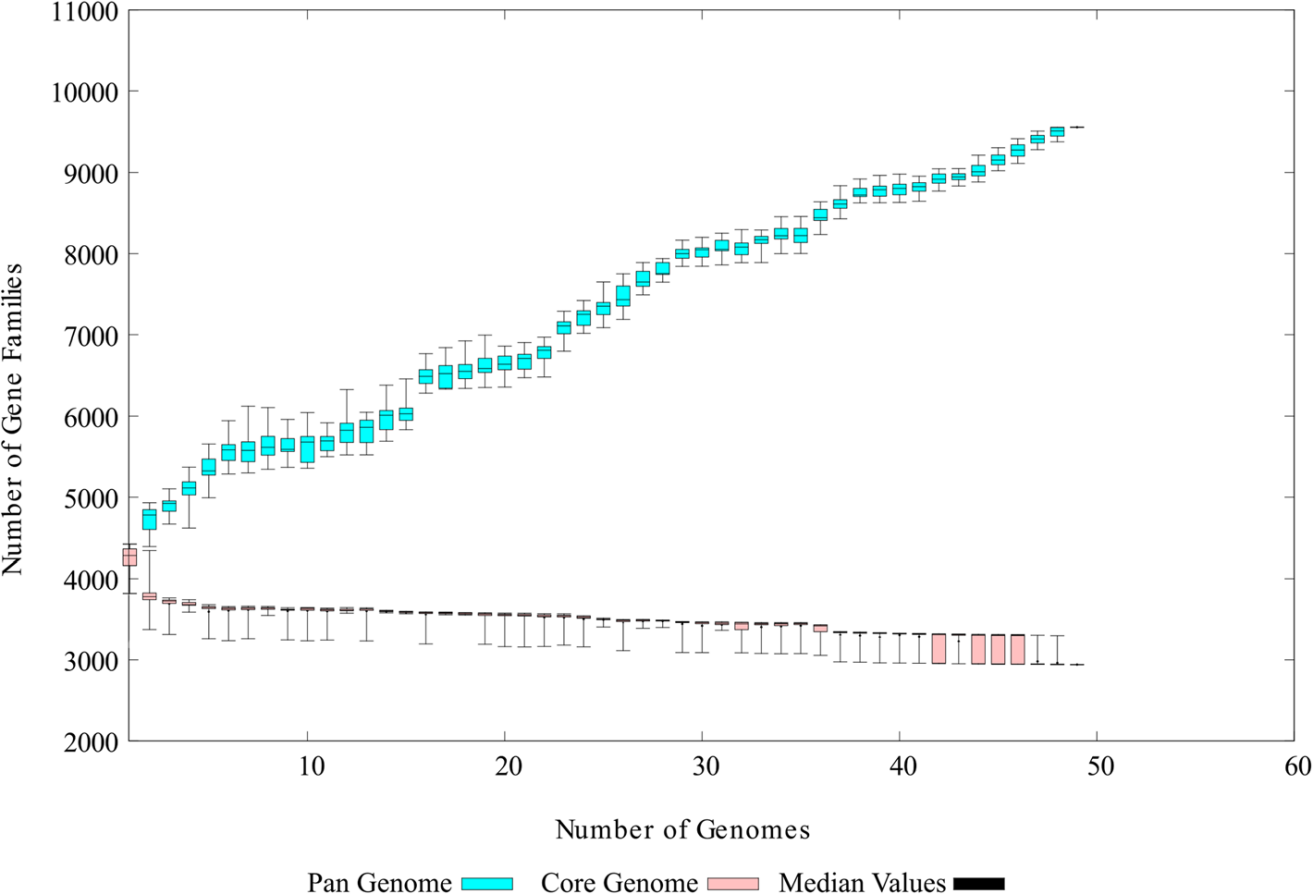
Pan-genome versus core genome plot to indicate the openness and closeness of the valid 49 *A. hydrophila* strains. The pan-genome increased continuously due to the addition of gene families, while the core genome stabilized

After identification of the 13 mislabelled strains, the remaining 49 strains were considered as valid members of the *A. hydrophila* pan-genome (Fig. 3). These 49 strains were included in further comparative genomic analyses. A total of 217,923 genes were predicted by Prodigal software (Oak Ridge National Laboratory, USA) across all 49 genomes. There was an average of 4448 genes present in each genome. The low quality of the draft genomes could be the cause of this small overestimation. These genes were clustered into 9560 homologue gene clusters (HGCs), which constitute the pan-genome of *A. hydrophila*. A core genome of 2942 HGCs was predicted (Additional file 1). The remaining 49 strains were clustered into different clades. In the reanalysis, a pan-genome matrix was generated on the basis of the absence or presence of genes across all the strains. The phylogenetic relationship of strains did not differ much in comparison to the first analysis (Fig. 3). A comparison of the pan-genome and the core genome based on the progress of the clustering algorithm is shown in Fig. 4. With the addition of genomes, the pan-genome, found to be an open genome, continued to increase, while the core genome decreased initially but stabilized after the addition of a few genomes. Initially, complete genomes were added to avoid any unusual decrease or increase that could occur with the addition of draft genomes. Across all 49 genomes, 1254 ± 144 accessary genes were present, on average. The maximum number of accessary genes was shared by the NJ-35 genome, i.e., 1472 genes. Unique genes were found to be highly variable among the strains as there was no clear pattern. There was a total of 2981 unique genes present in this pan-genome. The highest number of unique genes (*n* = 298) was found in the WCHAH045096 strain.

### Functional annotation

**Fig. 5:**
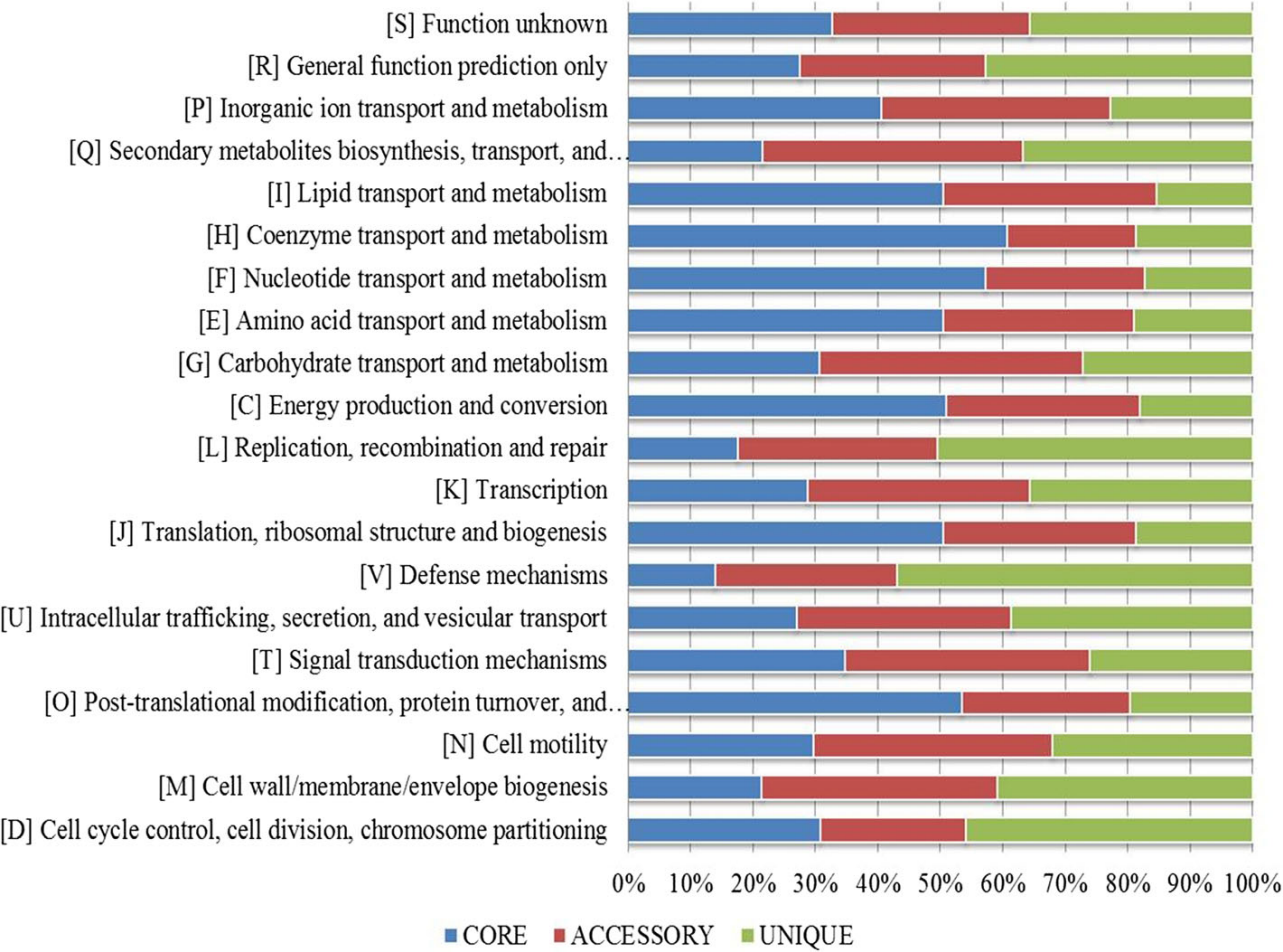
COG-based functional classification of core, accessary and unique genes. The distribution of functional genes among these core genes represents the abilities of all the strains in this pan-genome, whereas the remaining dispensable genes provides of increasing trend of genes

**Fig. 6:**
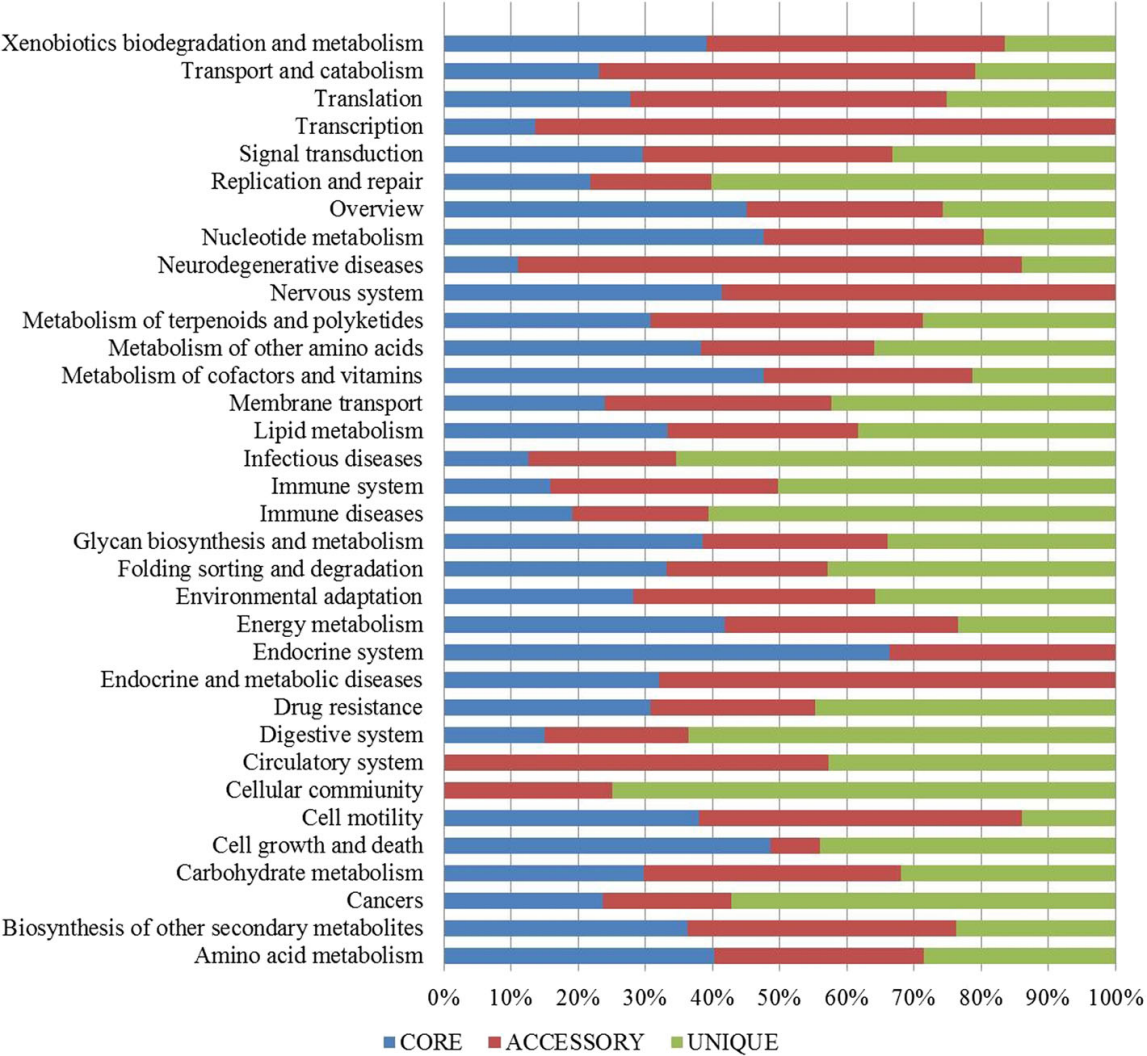
KEGG-based functional annotation of all the protein datasets of the core, accessary and unique genes. Different classes of genes are found among the strains

The datasets of core and dispensable genomes were extracted and locally aligned against the Cluster of Orthologous Group (COG) and Kyoto Encyclopedia of Genes and Genomes (KEGG) databases using the Usearch algorithm. Identification of COG functional categories revealed that the core genome was enriched in the metabolism and posttranscriptional modification classes (Fig. 5), while the accessary genes were highly enriched in secondary metabolite biosynthesis and catabolism. The unique proteins were mostly found in the replication, defence mechanisms, cell wall biogenesis and cell cycle control classes (Fig. 5). In the KEGG functional annotation (Fig. 6), core genes were found to be higher among the classes of substance dependence, amino acid metabolism, cell cycle and endocrine system. Accessary genes were enriched among the transcription and metabolic diseases classes. Among the unique proteins, more proteins were found to be involved in cell signalling, cellular community and infectious diseases.

### Comparison of *A. hydrophila* strains with ST-251 and other STs

**Fig. 7:**
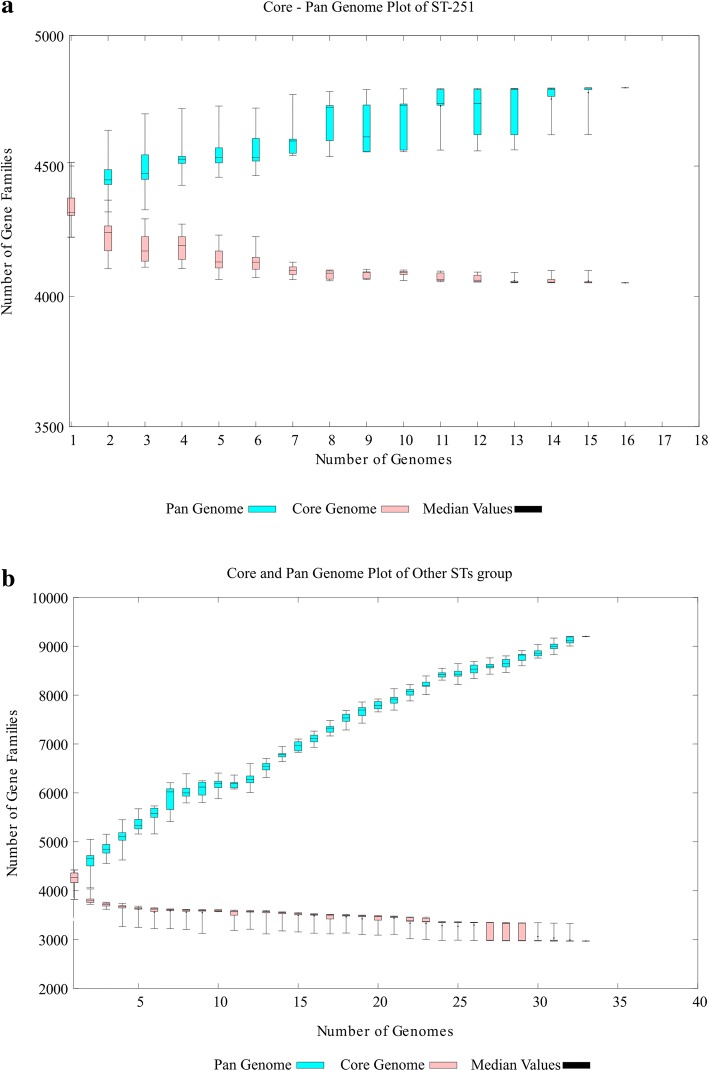
Predictions of the core genome and pan-genome of the ST-based groups. **a** Prediction of the core and pan-genome of the *A. hydrophila* strains with ST-251 and (**b**) other STs. ST-251 represents a closed pan-genome, while the other STs represent an open pan-genome

The genomes of *A. hydrophila* strains with ST-251 and other STs were also compared and analysed to gain further insights. Among strains in the ST-251 group (*n* = 16), the core genome contained 4052 genes with 272 ± 48 and 289 genes in accessory and unique fractions, respectively (Additional file 2). In contrast, among strains from the other STs group (*n* = 33), the core genome was composed of 2968 genes with 1165 ± 137 and 2936 genes in accessory and unique fractions, respectively (Additional file 3). Based on these results, it is predicted that the ST-251 group has a closed pan-genome whereas the other, more diversified ST groups have an open pan-genome (Fig. 7).

**Fig. 8:**
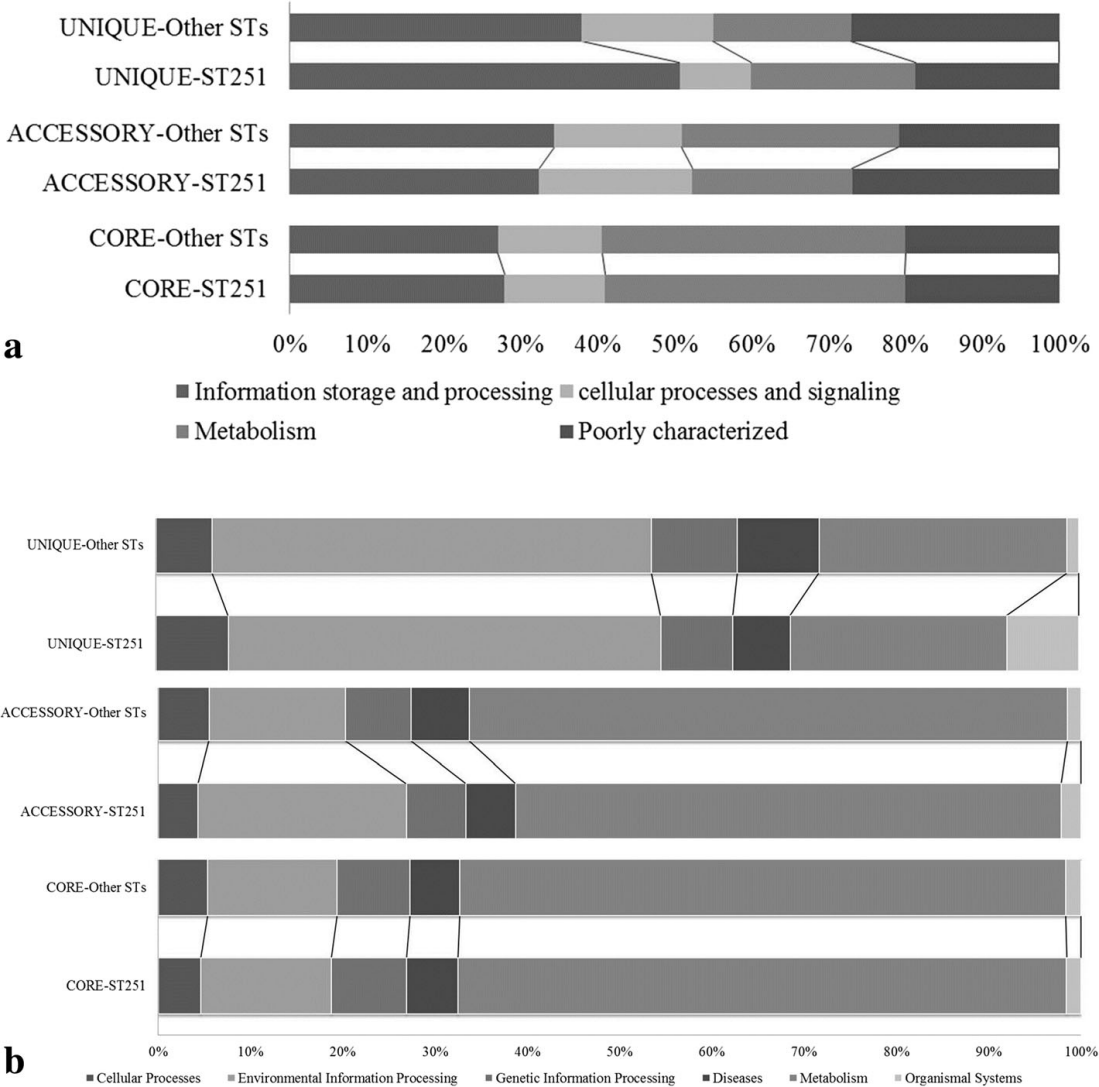
Comparison of functionally annotated proteins among the ST-251 and other ST groups. **a** Comparison of *A. hydrophila* strains with ST-251 and other STs based on COG functional categories. Unique and accessary classes are different, while there is little difference between the core genes. **b** Distribution of KEGG superfamilies among the various gene fractions of ST-251 and the other STs

COG superfamily functional categories revealed that unique fractions in ST-251 were higher in information storage processing and metabolism-related functional families than the other STs, whereas cellular processing and poorly characterized gene functional families were higher in the other STs group. The accessory gene fraction was the reverse of the above mentioned unique fraction in both categories for ST-251 and the other STs group. Within both groups, the core genome was comparatively similar (Fig. 8). KEGG-based functionally annotated superfamilies revealed results very similar to those inferred from the COG database. The ST-251 group had a higher number of unique genes among the cellular processes in comparison to other superfamilies compared to the other STs group. The accessory gene fraction in ST-251 had more genes in environmental information processing, whereas all the remaining superfamilies, such as cellular processes, genetic information processing, metabolism, diseases and organismal systems, were dominate in the other STs group. Similar to the COG results, the core genomes of both groups were equal in superfamilies.

### The comparative analysis of genome elements

#### Prophages

**Fig. 9:**
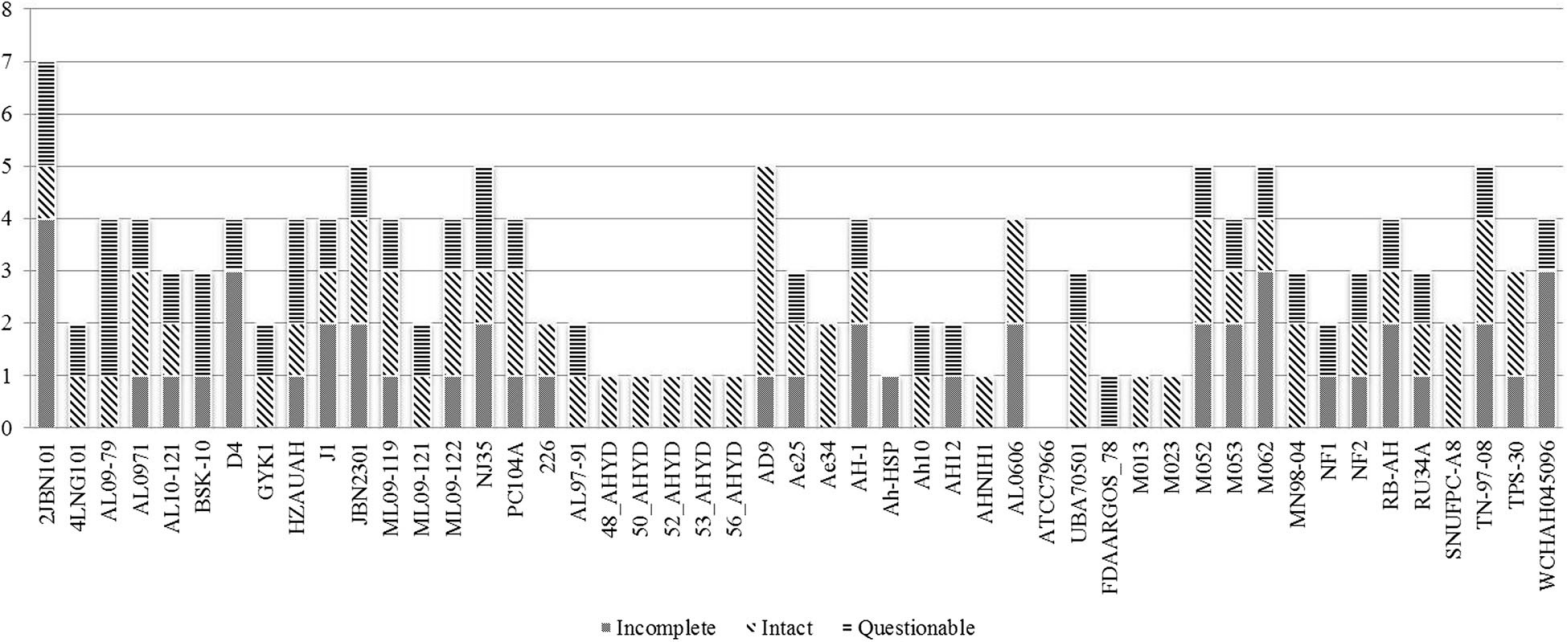
Number of prophages with their completeness profiles in *A. hydrophila* strains. Only the ATCC-7966 strain did not have any prophages, while the 2JBN101 strain had 7 prophages

**Fig. 10:**
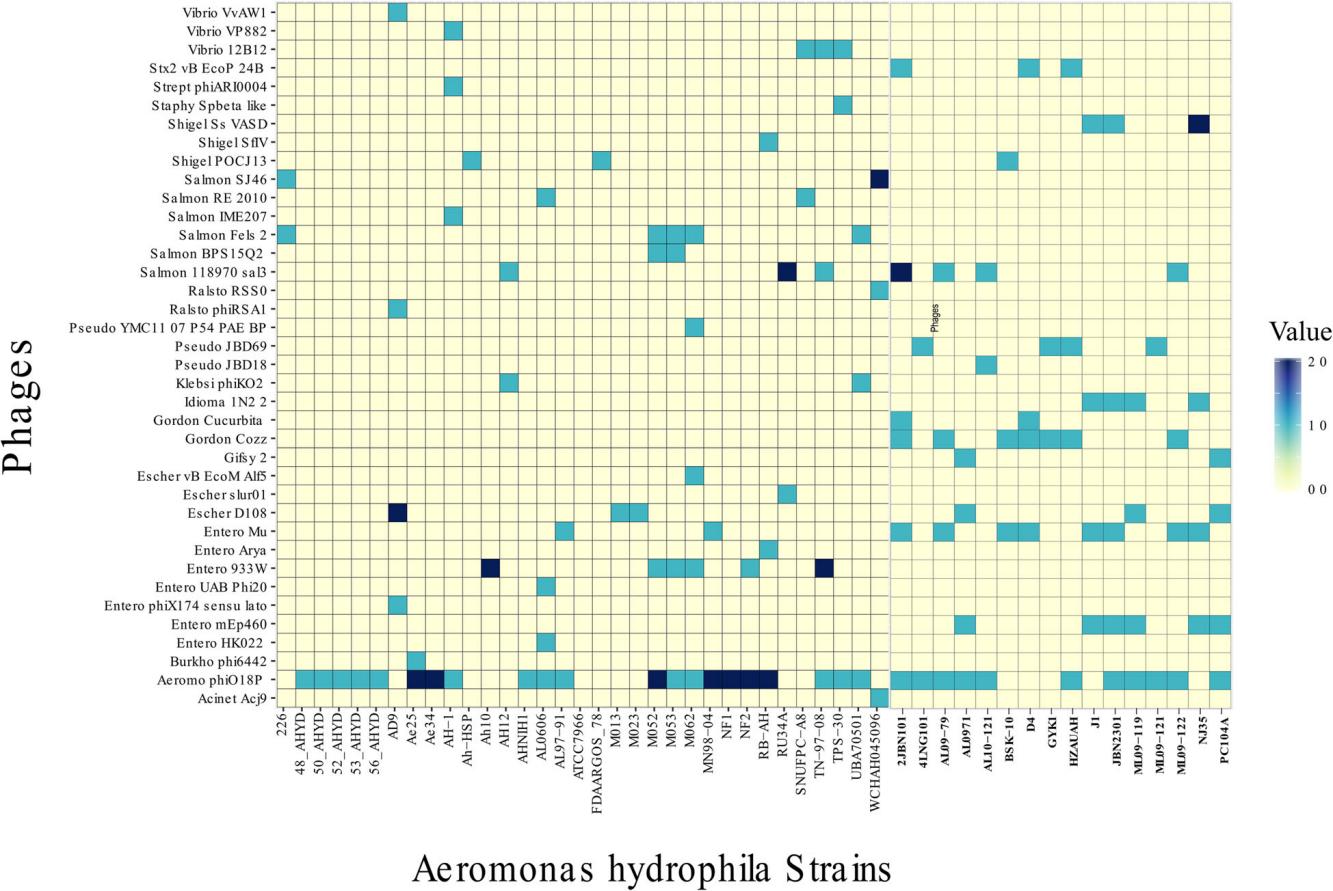
Type of prophages present across the *A. hydrophila* strains. Multiple prophages were detected in different strains. A maximum of two phages belonging to one prophage type were detected within one strain

Prophages were detected using the PHAST server across all the genomes. A total of 143 prophages were detected (Fig. 9). Among these, there were 57 intact phages (completeness score was above 90), while 39 were questionable (completeness score was 60–90), and 47 were incomplete phages (completeness score was less than 60). On average, there were approximately 2 prophages present in each strain. The maximum number of phages (*n* = 7) was detected in strain 2JBN101, whereas no prophages were found in the ATCC7966 strain. Among these 143 prophages, there were 38 types of prophages present (Fig. 10). Aeromonas phi O18P was highly distributed among the *A. hydrophila* strains regardless of their ST groups. As expected, there was much more variety of phages among the other STs group compared to ST-251 group. Of greater concern is the presence of shiga toxin-converting bacteriophages, such as 933 W and Stx2. An intact enterobacter 933 W prophage was detected in Ah-10 (other STs group), whereas Stx2 vB EcoP 24B phage was found in the strains 2JBN101, D4 and HZAUAH (ST-251). All the strains carrying shiga toxin bacteriophages were isolated from fish.

#### Antibiotic resistance genes

**Fig. 11:**
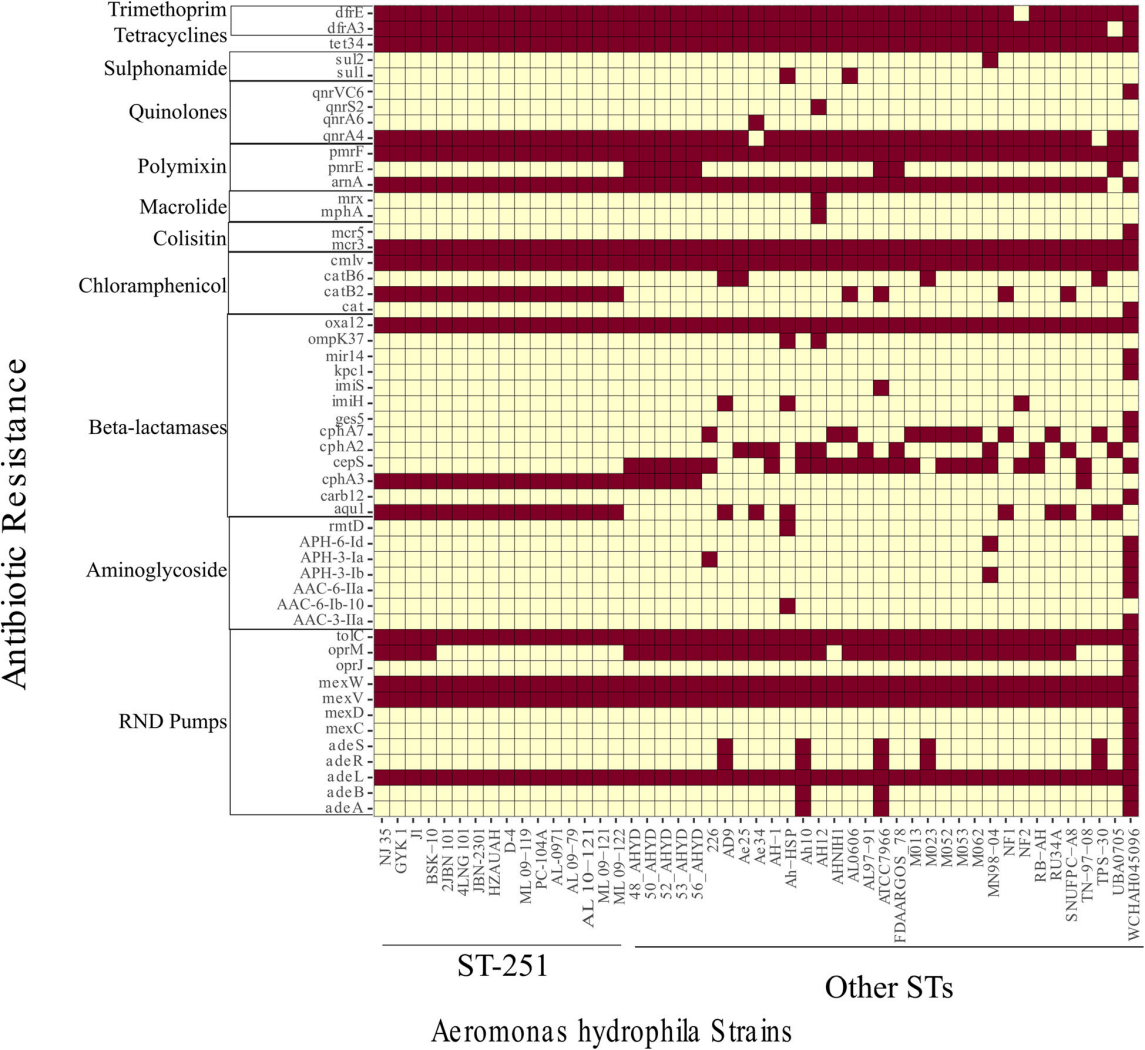
Antibiotic resistance genes and their distribution across the *A. hydrophila* strains. The red colour represents the presence of that particular gene from that antibiotic class in the strain, while the light yellow colour represents the absence that gene

More than two antibiotic resistance genes were found across all the genomes in this pan-genome (Fig. 11). Almost all the strains have one or more genes related to beta-lactamases, aminoglycosides, colistin and polymyxins, tetracycline, and chloramphenicol. Among the various types of multidrug efflux pumps found in microbial genomes, resistance nodulation cell division (RND) pumps are very important. Here, this study found that the presence of outer membrane proteins, such as OprM, OprJ, OmpK and tolC, was functionally related to antibiotic resistance. In addition, the quinolone resistance gene qnrA4 was found in almost all the strains. All the ST-251 strains had almost the same antibiotic resistance patterns, except that strains NJ35, GYK1, BSK-10 and J1 had oprM, while the other members lacked this outer membrane protein, indicating the same origin and propagating environments. In contrast, the diverse other STs group had different ranges of antibiotic resistances. Among these strains, WCHAH045096 had resistance genes from almost all the classes. This particular strain was isolated from a hospital waste drain, exhibiting clear evidence of horizontal gene transfer.

#### Virulence factors

**Fig. 12:**
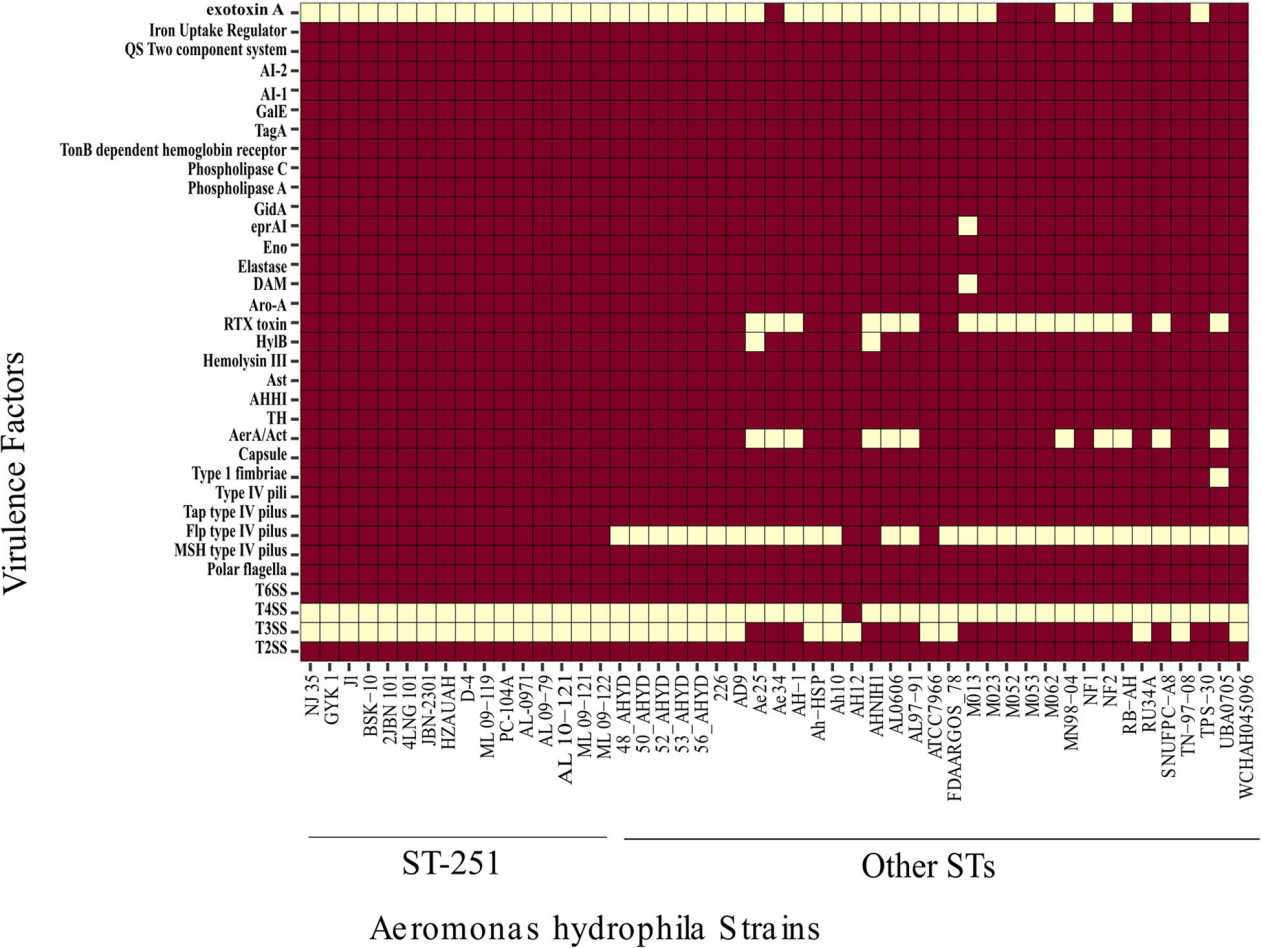
Distribution of virulence factors across all 49 genomes and among the various ST groups. The red colour represents the presence of the gene, and the light yellow colour represents the absence of the gene

The Virulence Factors database was screened in this study to determine the distribution of various virulence factors among these 49 strains (Fig. 12). The major virulence factors found among all the strains were secretion systems, the motility and adhesion system, the quorum sensing system and toxins. For secretion systems, T2SS and T6SS were commonly found, while T3SS and T4SS were observed less in *A. hydrophila* for clinical isolates of both human and fish origin. The RTX toxin-producing genes were found in all those strains that were not harbouring the T3SS genes. There was a good difference in the virulence factors among the ST-251 and other ST groups. With the exceptions of T3SS, T4SS and exotoxin A, all the virulence factors investigated were present in the ST-251 group, whereas the virulence factors were more varied in the other STs group. Among the other STs’ strains, Flp type IV pili and aerolysin (AerA) were mostly found, while the presence of T3SS and exotoxin A showed the dynamic behaviour of this group.

## Discussion

*Aeromonas* species are highly dynamic in nature [3]. Therefore, their classification has remained an unsolved riddle. Pathogenically important *A. hydrophila* is widely present in aquatic sources and causes disease in both human and fish [16]. Because of its global presence, there is a high number of strains reported. This distribution also led to the intraspecific variation and diverse behaviour in strains that could be due to horizontal gene transfer and mutations. Hence, ambiguous classification can lead to misclassification [8]. In this confusing situation, WGS has been helpful for allowing us to investigate the whole genome and classify them accordingly. Additionally, in silico tools have provided the opportunity to determine the differences between strains and to identify the epidemiologically important strains [10]. In this study, identification of core genes and estimation of genetic variation were performed among the 62 publicly available *A. hydrophila* genomes. By utilizing in silico tools to compare these genomes, how these genomes fit into the presented data was discussed. Prominent results that may form the basis of future classification of *A. hydrophila* were also observed.

In this study, phylogenomic analysis demonstrated that there was wide diversification among the 62 genomes. Moreover, there are many possible distinct species that are confused with *A. hydrophila* and incorrectly classified. This study identified such discrepancies and marked the distinction between valid *A. hydrophila* and incorrectly classified species. In silico tools, such as core genome-based phylogeny, ANI variation and genome-to-genome distance calculations have been proved helpful in identifying the correct classification. The results of this study are validated, as two of the previously misclassified genomes, SSU and BWH65, were also included, and those genomes were classified separately from valid *A. hydrophila* species. In a previous study performed by Beaz-Hidalgo et al. [8], ANI has been used to discriminate the mislabelled strains, but the distinction was not clear and robust. Core genome-based phylogeny is a relatively new proposed method of classification of genomes, which is consistent with MLST in other studies [10, 14]. As previously reported, ANI and dDDH calculations could confirm these variations [15]. In ANI variation and dDDH analyses, when compared with the reference genome ATCC7966, all the core genomes showing variation were already classified into different phylogenetic clusters.

Bacterial genomes are much more flexible in comparison to eukaryotes. Pan-genomic studies are always performed to examine the varying genetic structures present among different individual bacteria strains of a species [17]. In our study, closely related strains of the same species are also diverse in nature. The variation in terms of encoded proteins among the strains is possibly correlated to geographical isolation and distinct lifestyles, as has been reported in previous studies [18]. Genome comparison further revealed that the dispensable genome truly explained the interstrain variation. These varying fractions include a wide range of genes that could explain the impact of exposure to the environment on the various strains [19]. For instance, environments bearing antibiotic resistant bacteria may eventually transfer the resistance genes to non-resistant bacteria [20]. This scenario could explain why the strain WCHAH045096 harbours the highest number of unique genes, most of which are antibiotic resistance genes. Higher numbers of accessary genes, as in the case of strain NJ-35 in this study, also inferred the presence of shared genes that are prevalent and important for that strain [5]. Based on these fractions, varying patterns and introductions of genes could be estimated, which can be helpful in designing epidemiological strategies and in understanding the changing behaviour of *A. hydrophila*.

Codon usage and amino acid usage patterns may also show interstrain variation and can reveal the molecular basis of evolution of *A. hydrophila* [21]. Exploring these parameters may also reveal the type of natural selection pressure on genes, as found in previous studies [22]. An initial analysis of amino acid frequency of all the genes was performed using BioEdit and CodonW software packages [23, 24]. It revealed significant differences in certain amino acids, such as methionine (Met), leucine (Leu), and alanine (Ala), among the core and dispensable genes (Additional file 4), while different amino acids, such as cysteine (Cys), proline (Pro), arginine (Arg), serine (Ser) and tryptophan (Trp), were found to be significantly frequent in the ST-251 group compared to the other STs group. Although significant differences among the amino acids of different groups exist, we do not know the implications of this difference in the *A. hydrophila* pan-genome. In the future, a thorough codon usage analysis followed by multiple correspondence analyses (MCA) among members of the *A. hydrophila* pan-genome may broaden our understanding regarding selection pressure, point mutations and amino acid conservation.

The functional annotation of COG and KEGG databases to infer the functions of proteins is also of great concern [15, 25, 26], as they categorize the varying fractions into families and superfamilies. Based on both COG and KEGG database assignment results, the core genome was enriched in metabolism-related genes, while dispensable genes were found to be higher among the motility, cell communication and defence mechanisms. Surprisingly, both databases produced almost similar results. In previous studies, Bhardwaj and Somanshi [27] found the same results in pan-genomic studies, while in some studies, both databases did not show similar results because KEGG has more metabolism-related genes [25, 28]. This functional annotation can help in the overview of the distribution of proteins and their relation to interstrain variation.

In this study, all of these genomes were also locally aligned against different databases including phages, antibiotic resistance genes and virulence factors. Phages are of prime importance in impacting bacterial evolution as they are responsible for loci rearrangements and deletions. Approximately 70% of aquatic bacteria are infected with prophages [29]. A higher number of phages are of great concern as they can turn an avirulent strain into a virulent one [30]. As an aquatic bacterium, *A. hydrophila* also harbours multiple phages in its genome. Like other bacteria, the distribution of prophages in *A. hydrophila* is not homogenous. Our study documented the presence of bacteriophages across all the genomes and quantified them. Moreover, the presence of the prophages carrying the shiga toxin gene showed that there is a chance of possible outbreaks related to severe gastrointestinal disorders. Investigation of prophage function is beyond the scope of this study. However, it would be fascinating to elucidate a specific pattern between phage occurrence and bacterial virulence.

Mobilome-based features, such as virulence factors and antibiotic resistance genes, are very important in interstrain variation as well as pathogenicity. Moreover, virulence factors have also been proposed as the bases of classification systems. As similar bacterial species have conserved virulence factors [3], this study also showed that T2SS and T6SS were conserved among the *A. hydrophila* strains, whereas the occurrence of T3SS was variable among the strains. T3SS has a history of virulence in humans, and the data also showed that strains with T3SS were potentially zoonotic [31]. RTX toxin is also important for human gastrointestinal diseases as it can increase cell rounding and apoptosis [32]. This study also detected the presence of all the important virulence factors regardless of their genetic components. In previous studies, the virulence factors investigated here have been found to be important for bacterial pathogenicity [3, 33].

Antibiotic resistance was extensively investigated in *A. hydrophila* strains worldwide. Beta-lactamase resistance has already been widely reported [34]. Colistin and polymyxin resistances have also been established for *Aeromonas* spp. [35, 36]. In this study, the presence of multiple resistance genes associated with the three antibiotics supported the previous reports. Additionally, RND pumps are key players in multiple drug resistance and have different types of components that efflux the several antibiotic molecules [37]. We found that several genes encoding RND efflux pumps, including *tolC*, *mexW*, *mexV* and *adeL*, are present in the chromosomes of all the strains. This implies the possibility that the *A. hydrophila* strains investigated here have acquired multiple resistances to many antimicrobials. Moreover, it should be noted that almost all the strains have quinolone resistance gene *qnrA4*, whereas the other *qnr* genes are rare. We cannot determine whether an association between *qnrA4* and quinolone resistance exists, but it might be interesting to evaluate whether the gene contributes to quinolone resistance. There were no resistance genes found against sulphonamide and aminoglycosides. It is speculated that sulphonamides and aminoglycosides could be a possible component of treatment regimens, although further investigation is needed.

As for the ST group-based comparisons, there was more diversity between the two groups. However, the least variation was in ST-251, although the reported strains were from different continents and countries. This could be possibly owing to the movement of diseased hosts (either fish or human) or loose quarantine practices between countries, as has been previously described [38]. Among the other STs group, substantial variation in virulence factors, antibiotic resistance and phages was found. This diversity is the evidence of the changing behaviour of the bacteria across time and environmental conditions.

## Conclusion

Application of comparative genomics to *A. hydrophila* provides insights into taxonomic relationships among these strains. There are also some strains that are mislabelled and should not be regarded as *A. hydrophila* for future analysis. Further, downstream analysis indicated that due to environmental conditions, this bacterium is an emerging potential zoonotic pathogen. Based on the presence or absence of genes, the calculated interstrain relationships are considerable for future analyses. Regardless of their strain typing category, a large number of prophages were found in the pan-genome, especially Aeromonas phi 018 present in multiple strains. Various virulence factors, including T3SS and T6SS, were found in this pan-genome. However, a higher number of virulence factors were detected in ST-251 compared to other STs group. Similarly, various antibiotic resistance genes from different antibiotic classes were found in this pan-genome, such as beta-lactamases, colistin, and polymyxin, indicating the multiple drug resistance ability of this bacterium. Moreover, a specific combination of virulence factors and antibiotic resistance genes might increase the bacterial virulence. Downstream analyses involving proteome analyses could reveal more insights into these factors.

## Methods

### Genomes, genome properties and gene predictions

All publicly available *A. hydrophila* genome sequences (65 in total: 14 full genomes, 51 draft genomes) at the time of this study were obtained from the National Center for Biotechnology Information (NCBI Resource Coordinators, 2016). The properties of the complete and draft genomes included in this study are summarized in Table 1. Due to odd behaviour in protein prediction and alignment, draft sequences of 3 strains (ARKANSAS 2010, ATCC7966–2, and CIP-107985) were not included. To differentiate two genomes with the same name, AH-1, one of the genomes was renamed to AH-12. G + C % calculations were performed using BioEdit v 7.6.2 [23]. Genome alignment in MAUVE v.20150226 was performed to refine the genome assemblies and genome scaffolding [39]. Protein predictions were performed using Prodigal software (Oak Ridge National Laboratory, USA) [40].

**Table 1:** All the valid 49 strains of *A. hydrophila* included in this study

Strain	Source	Country	Accession	Base pairs (Mbp)	GC%	Reference
ATCC7966	Fishy milk	USA	NC_008570	4.74	61.50	[50]
AH10	Grasscarp	China	NZ_CP011100	4.91	61.10	
AL0606	Goldfish	USA	NZ_CP010947.1	4.90	61.37	[51]
AL0971	Channel catfish	USA	NZ_CP007566	5.02	60.80	[52]
D4	Fish	China	NZ_CP013965	5.28	60.46	
GYK1	Missing	China	NZ_CP016392	4.95	60.80	
J1	Diseased carp	China	NZ_CP006883	5.00	60.90	[5]
JBN2301	Crucian Caro	China	NZ_CP013178	5.15	60.78	[53]
ML09–119	Diseased catfish	USA	NC_021290	5.02	60.80	[54]
NJ35	Diseased carp	China	NZ_CP006870	5.28	60.50	[5]
PC104A	Pond soil	USA	NZ_CP007576	5.02	60.80	[55]
AHNIH1	Human	USA	NZ_CP016380	5.05	61.07	[56]
2JBN101	Crucian Carp	China	LXME00000000	5.09	60.80	
WCHAH045096	Sewage	China	PDWA00000000	5.12	61.00	
4LNG101	Fish	China	MJGY00000000	4.99	60.10	
UBA705	Environment	Australia	DBMF00000000	4.25	61.60	[57]
48_AHYD	Human	USA	JVFM00000000	4.70	61.60	
50_AHYD	Human	USA	JVES00000000	4.67	61.60	
52_AHYD	Human	USA	JVDW00000000	4.68	61.60	
53_AHYD	Human	USA	JVDL00000000	4.67	61.60	
56_AHYD	Human	USA	JVCD00000000	4.68	61.60	
226	Human Urine	Malaysia	JEML00000000	5.11	60.90	
AD9	Wetland Sediment	USA	JFJO00000000	4.91	61.30	[58]
Ae25	Koi Carp	Sri Lanka	BEYT00000000	4.76	61.30	[59]
Ae34	Koi Carp	Sri Lanka	BAXY00000000	4.71	61.60	[60]
AH-1	Fish	Canada	LYXN00000000	4.76	61.40	
AH-1(2)	Patient	USA	LSZC00000000	5.12	60.90	
Ah-HSP	Human Blood	Brazil	MTPO00000000	5.03	61.18	
AL09–79	Catfish	USA	LRRV00000000	4.97	60.90	[61]
AL10–121	Catfish	USA	LRRW00000000	4.97	60.90	[61]
AL97–91	Tilapia	USA	LYZF00000000	4.83	61.19	[62]
BSK-10	Fish	China	NBOV00000000	4.96	61.00	[63]
FDAARGOS_78	Human Stool	USA	JTBD00000000	4.93	61.00	
HZAUAH	Missing	China	MRDF00000000	5.04	60.90	[64]
M013	WaterFall	Malaysia	JRWS00000000	4.97	61.00	
M023	WaterFall	Malaysia	JSWA00000000	4.91	60.90	[65]
M052	Water	Malaysia	MAKI00000000	4.97	61.10	
M053	Water	Malaysia	MAKJ00000000	4.96	61.10	
M062	WaterFall	Malaysia	JSXE00000000	4.97	61.10	
ML09–121	Catfish	USA	LRRX00000000	4.97	60.90	[61]
ML09–122	Catfish	USA	LRRY00000000	4.97	60.90	[61]
MN98–04	Tilapia	USA	LYZG00000000	4.88	61.10	[62]
NF1	Human	USA	JDWC00000000	4.81	61.10	[66]
NF2	Human	USA	JDWB00000000	4.79	61.30	[66]
RB-AH	Soil	Malaysia	JPEH00000000	5.09	60.80	
RU34A	Missing	USA	FTME00000000	4.83	61.20	
SNUFPC-A8	Salmon	South Korea	AMQA00000000	4.97	60.80	[67]
TN-97-08	Diseased Bluegill	USA	LNUR00000000	5.09	60.80	[68]
TPS-30	Fish	China	NBWY01000000	4.93	61.20	

### In silico typing, average nucleotide identity and genome-to-genome distance calculation

To identify the strain typing based on loci of housekeeping genes, in silico MLST was performed utilizing the CGE webserver (https://cge.cbs.dtu.dk/services/MLST-1.8/) [41]. As in silico MLST had very few registered loci profiles, for this purpose, bi-directional similarity estimation with the *A. hydrophila* ATCC7966 reference genome was performed utilizing the OrthoANIu tool v 1.2 to identify two-way average nucleotide identity (ANI) values [11]. To further confirm the results of MLST and ANI, dDDH was also performed utilizing the genome-to-genome calculator (http://ggdc.dsmz.de) [42]. The results of genome-to-genome distances were recorded according to recommended formula 2.

### Comparative genomic analysis

All the strains based on the ANI and dDDH acceptable results were included in the pangenome (core genome and dispensable genome) analysis using the BPGA algorithm [25]. Usearch was employed for clustering the homologous genes present in the pan genome [43]. Genes shared by all the strains were considered as the core genome, while the dispensable genes either present in two or more strains (accessary proteins) or present in only one strain (unique proteins) were also identified. The core genome plot was based on plotting the total number of shared genes with each subsequent addition of a genome against the number of genomes. The pangenome plot was based on plotting the total number of distinct gene families identified with the addition of each genome and number of genomes. Obtained representative sequences of core and pan genomes were used for functional annotations and further downstream analyses.

The core genome phylogeny was constructed on the basis of conserved genes among all the strains. Core genes were aligned using MUSCLE [44]. To generate a neighbour joining tree, concatenated aligned sequences were utilized in Phylip, and MEGA v 6 was utilized to visualize the phylogenetic tree [44]. Phylogenetic trees were further smoothed using the iTOL tree website (http://itol.embl.de/) [45]. The phylogeny based on the presence/absence matrix was generated by the R statistical language base functions and heatmap function.

### Functional annotations

Identified proteins were further functionally annotated against Cluster of Orthologous Group (COG) and Kyoto Encyclopedia of Genes and Genomes (KEGG) databases [46]. Integrated prophages were identified using the PHAST server (http://phast.wishartlab.com/index.html) [47]. To identify the prevailing antibiotic resistance genes and virulence factors, genomes were aligned utilizing Ublast with an e-value of 1 × 10^− 6^ and alignment length of 80% [43] against the Comprehensive Antibiotic Resistance Database (CARD) (https://card.mcmaster.ca/) [48] and the Virulence Factors Database (VFDB, www.mgc.ac.cn/VFs/main.htm) [49]. Further, aligned sequences were manually checked for the annotated features to the database and NCBI. All the graphical figures and heat-map charts were generated by R package ggplot2.

### Comparison of the genome sequences of *A. hydrophila* with different sequence types (STs)

Our previous study demonstrated that *A. hydrophila* ST-251 is a high-risk type in fish farms [5]. To determine whether there exists a difference in genome size and composition between ST-251 and other STs, a comparative genome analysis was re-run on these categories separately to compare the core and dispensable genes prevalent among both groups (Additional files 2 and 3). Similarly, the functional annotations based on COG and KEGG databases were reanalysed based on these categories.

## Additional files

Additional file 1: Number of core, accessory and unique genes among the valid 49 strains of *A. hydrophila*. (DOCX 19 kb)

Additional file 2: Number of core, accessory and unique genes among the 16 strains of the ST-251 group. (DOCX 15 kb)

Additional file 3: Number of core, accessory and unique genes among the 33 strains of the other ST groups. (DOCX 17 kb

Additional file 4: Amino acid frequency of all 49 strains belonging to *Aeromonas hydrophila*. Comparison of core and dispensable genomes on the basis of all 20 amino acids found in the *A. hydrophila* pan-genome. (DOCX 16 kb)

## Abbreviations

AerA: Aerolysin
ANI: Average nucleotide identity
CARD: Comprehensive Antibiotic Resistance Database
cgMLST: Core genome MLST
COG: Cluster of Orthologous Group
dDDH: Digital DNA-DNA hybridization
HG: Hybridization groups
HGCs: Homologue gene clusters
KEGG: Kyoto Encyclopedia of Genes and Genomes
MLST: Multiple locus sequence type
NCBI: National Center for Biotechnology Information
RND: Resistance nodulation cell division pumps
ST: Sequence type
T3SS: Type III secretion system
T4SS: Type IV secretion systems
T6SS: Type VI secretion systems
VFDB: Virulence Factors Database
WGS: Whole genome sequencing
