# Supplementary material for: Comparative genome analysis provides deep insights into Aeromonas hydrophila taxonomy and virulence-related factors
Source: BMC Genomics. 2018 Sep 26;19:712. doi: 10.1186/s12864-018-5100-4 (PMC6158803; doi:10.1186/s12864-018-5100-4)
Supplement: Supplementary file 1 — Number of core, accessory and unique genes among the valid 49 strains of A. hydrophila. (DOCX 19 kb) [file 12864_2018_5100_MOESM1_ESM.docx]

Additional file 1: No. of Core genes, accessory genes and unique genes among all the valid 49 strains of *A. hydrophila*.

| Strains | Source | No. of core genes | No. of accessory genes | No. of unique genes |
| --- | --- | --- | --- | --- |
| ATCC7966 | Environment | 2942 | 1069 | 53 |
| AH10 | Fish | 2942 | 1153 | 68 |
| AL0606 | Fish | 2942 | 1211 | 82 |
| AL0971 | Fish | 2942 | 1359 | 1 |
| D4 | Fish | 2942 | 1414 | 3 |
| GYK1 | Fish | 2942 | 1253 | 17 |
| J1 | Fish | 2942 | 1346 | 6 |
| JBN2301 | Fish | 2942 | 1449 | 0 |
| ML09-119 | Fish | 2942 | 1359 | 0 |
| NJ35 | Fish | 2942 | 1472 | 82 |
| PC104A | Environment | 2942 | 1363 | 0 |
| AHNIH1 | Human | 2942 | 1112 | 104 |
| 2JBN101 | Fish | 2942 | 1453 | 0 |
| WCHAH045096 | Environment | 2942 | 1165 | 298 |
| 4LNG101 | Fish | 2942 | 1337 | 45 |
| UBA705 | Environment | 2942 | 708 | 168 |
| 48_AHYD | Human | 2942 | 1107 | 1 |
| 50_AHYD | Human | 2942 | 1108 | 5 |
| 52_AHYD | Human | 2942 | 1107 | 2 |
| 53_AHYD | Human | 2942 | 1101 | 5 |
| 56_AHYD | Human | 2942 | 1101 | 4 |
| 226 | Human | 2942 | 1287 | 189 |
| AD9 | Environment | 2942 | 1202 | 138 |
| Ae25 | Fish | 2942 | 1174 | 103 |
| Ae34 | Fish | 2942 | 1061 | 70 |
| AH-1 | Fish | 2942 | 1370 | 110 |
| AH12 | Human | 2942 | 1081 | 140 |
| Ah-HSP | Human | 2942 | 1309 | 151 |
| AL09-79 | Fish | 2942 | 1352 | 0 |
| AL10-121 | Fish | 2942 | 1356 | 0 |
| AL97-91 | Fish | 2942 | 1292 | 5 |
| BSK-10 | Fish | 2942 | 1366 | 1 |
| FDAARGOS_78 | Human | 2942 | 1114 | 76 |
| HZAUAH | Missing | 2942 | 1435 | 2 |
| M013 | Environment | 2942 | 1188 | 210 |
| M023 | Environment | 2942 | 1205 | 133 |
| M052 | Environment | 2942 | 1402 | 4 |
| M053 | Environment | 2942 | 1399 | 8 |
| M062 | Environment | 2942 | 1407 | 14 |
| ML09-121 | Fish | 2942 | 1345 | 1 |
| ML09-122 | Fish | 2942 | 1354 | 3 |
| MN98-04 | Fish | 2942 | 1291 | 49 |
| NF1 | Human | 2942 | 1160 | 85 |
| NF2 | Human | 2942 | 1156 | 92 |
| RB-AH | Environment | 2942 | 1395 | 10 |
| RU34A | Missing | 2942 | 1136 | 112 |
| SNUFPC-A8 | Fish | 2942 | 1259 | 180 |
| TN-97-08 | Fish | 2942 | 1343 | 104 |
| TPS-30 | Fish | 2942 | 1277 | 47 |
