# Supplementary material for: Comparative genome analysis provides deep insights into Aeromonas hydrophila taxonomy and virulence-related factors
Source: BMC Genomics. 2018 Sep 26;19:712. doi: 10.1186/s12864-018-5100-4 (PMC6158803; doi:10.1186/s12864-018-5100-4)
Supplement: Supplementary file 2 — Number of core, accessory and unique genes among the 16 strains of the ST-251 group. (DOCX 15 kb) [file 12864_2018_5100_MOESM2_ESM.docx]

Additional file 2: No. of core genes, accessory genes and unique genes among the 16 strains of ST-251 group.

| Strain | No. of core genes | No. of accessory genes | No. of unique genes |
| --- | --- | --- | --- |
| AL0971 | 4052 | 265 | 1 |
| D4 | 4052 | 310 | 10 |
| GYK1 | 4052 | 150 | 25 |
| J1 | 4052 | 250 | 6 |
| JBN2301 | 4052 | 352 | 1 |
| ML09-119 | 4052 | 268 | 1 |
| NJ35 | 4052 | 282 | 179 |
| PC104A | 4052 | 270 | 0 |
| 2JBN101 | 4052 | 354 | 1 |
| 4LNG101 | 4052 | 223 | 57 |
| AL09-79 | 4052 | 258 | 0 |
| AL10-121 | 4052 | 262 | 0 |
| BSK-10 | 4052 | 268 | 1 |
| HZAUAH | 4052 | 338 | 2 |
| ML09-121 | 4052 | 251 | 1 |
| ML09-122 | 4052 | 255 | 4 |
