# Supplementary material for: Comparative genome analysis provides deep insights into Aeromonas hydrophila taxonomy and virulence-related factors
Source: BMC Genomics. 2018 Sep 26;19:712. doi: 10.1186/s12864-018-5100-4 (PMC6158803; doi:10.1186/s12864-018-5100-4)
Supplement: Supplementary file 3 — Number of core, accessory and unique genes among the 33 strains of the other ST groups. (DOCX 17 kb [file 12864_2018_5100_MOESM3_ESM.docx]

Additional file 4: No. of core genes, accessory genes and unique genes among the 33 strains of other ST group.

| Organism name | Core genes | Accessory | Unique |
| --- | --- | --- | --- |
| ATCC7966 | 2968 | 1039 | 55 |
| Ah10 | 2968 | 1125 | 70 |
| AL0606 | 2968 | 1178 | 86 |
| AHNIH1 | 2968 | 1087 | 104 |
| WCHAH045096 | 2968 | 1127 | 306 |
| 48_AHYD | 2968 | 1079 | 1 |
| 50_AHYD | 2968 | 1080 | 5 |
| 52_AHYD | 2968 | 1078 | 2 |
| 53_AHYD | 2968 | 1072 | 5 |
| 56_AHYD | 2968 | 1075 | 4 |
| 226 | 2968 | 1255 | 196 |
| AD9 | 2968 | 1176 | 137 |
| Ae25 | 2968 | 1142 | 107 |
| Ae34 | 2968 | 1033 | 73 |
| AH-1 | 2968 | 1346 | 110 |
| AH12 | 2968 | 1051 | 144 |
| Ah-HSP | 2968 | 1273 | 159 |
| AL97-91 | 2968 | 1267 | 5 |
| FDAARGOS_78 | 2968 | 1075 | 87 |
| M013 | 2968 | 1154 | 213 |
| M023 | 2968 | 1181 | 134 |
| M052 | 2968 | 1373 | 4 |
| M053 | 2968 | 1375 | 8 |
| M062 | 2968 | 1380 | 14 |
| MN98-04 | 2968 | 1264 | 49 |
| NF1 | 2968 | 1133 | 87 |
| NF2 | 2968 | 1127 | 93 |
| RB-AH | 2968 | 1369 | 9 |
| RU34A | 2968 | 1105 | 115 |
| SNUFPC-A8 | 2968 | 1216 | 196 |
| TN-97-08 | 2968 | 1313 | 107 |
| TPS-30 | 2968 | 1224 | 78 |
| UBA705 | 2968 | 677 | 173 |
