# Supplementary material for: Comparative genome analysis provides deep insights into Aeromonas hydrophila taxonomy and virulence-related factors
Source: BMC Genomics. 2018 Sep 26;19:712. doi: 10.1186/s12864-018-5100-4 (PMC6158803; doi:10.1186/s12864-018-5100-4)
Supplement: Supplementary file 4 — Amino acid frequency of all 49 strains belonging to Aeromonas hydrophila. Comparison of core and dispensable genomes on the basis of all 20 amino acids found in the A. hydrophila pan-genome. (DOCX 16 kb) [file 12864_2018_5100_MOESM4_ESM.docx]

Additional file 3: Amino acid frequency of all the 49 strains belonging to *Aeromonas hydrophila*. Comparison of Core and Dispensable genome on the basis of all the 20 amino acids found in *A.hydrophila* pangenome
